# Impact of vaccine economic programs on physician referral of children to public vaccine clinics: a pre-post comparison

**DOI:** 10.1186/1471-2458-6-7

**Published:** 2006-01-12

**Authors:** Richard K Zimmerman, Melissa Tabbarah, Janine E Janosky, Barbara Bardenheier, Judith A Troy, Ilene K Jewell, Barbara P Yawn

**Affiliations:** 1 Department of Family Medicine and Clinical Epidemiology, School of Medicine, University of Pittsburgh, Pittsburgh, PA, USA; 2 Department of Behavioral and Community Health Sciences, Graduate School of Public Health, University of Pittsburgh, Pittsburgh, PA, USA; 3 Health Services Research and Evaluation Branch, Immunization Services Division, National Immunization Program, Centers for Disease Control and Prevention, Atlanta, GA, USA; 4 Olmsted Medical Group, Department of Research, Rochester, MN, USA

## Abstract

**Background:**

The Vaccines for Children (VFC) Program is a major vaccine entitlement program with limited long-term evaluation. The objectives of this study are to evaluate the effect of VFC on physician reported referral of children to public health clinics and on doses administered in the public sector.

**Methods:**

Minnesota and Pennsylvania primary care physicians (n = 164), completed surveys before (e.g., 1993) and after (2003) VFC, rating their likelihood on a scale of 0 (very unlikely) to 10 (very likely) of referring a child to the health department for immunization.

**Results:**

The percentage of respondents likely to refer was 60% for an uninsured child, 14% for a child with Medicaid, and 3% for a child with insurance that pays for immunization. Half (55%) of the physicians who did not participate in VFC were likely to refer a Medicaid-insured child, as compared with 6% of those who participated (*P *< 0.001). Physician likelihood to refer an uninsured child for vaccination, measured on a scale of 0 to 10 where 10 is very likely, decreased by a mean difference of 1.9 (*P *< 0.001) from pre- to post-VFC. The likelihood to refer a Medicaid-insured child decreased by a mean of 1.2 (*P *= 0.001).

**Conclusion:**

Reported out-referral to public clinics decreased over time. In light of increasing immunizations rates, this suggests that more vaccines were being administered in private provider offices.

## Background

Although immunization rates for preschool children in the United States are high by historical standards, pockets of need exist and racial disparity occurs [1,2]. The Healthy People 2010 vaccination coverage goal for universally recommended vaccinations among children aged 19–35 months is 90% [3]. Economic barriers are among the barriers to achieving this goal. In 1992, a national study reported that more than 85% of physicians reported referring uninsured children to public vaccine clinics some of the time [4].

To address economic barriers, vaccine financing reforms, such as the Vaccines for Children Program (VFC), were developed and followed by a reduction in the proportion of physicians that refer children to public clinics for vaccination [5]. Begun in October 1994, the VFC provides states with free vaccines for children who are Medicaid-eligible, have no health insurance, or are Native Americans or Alaskan natives [6]. In addition, children whose insurance does not cover vaccines are eligible for VFC if they are vaccinated at a federally qualified health center or rural health clinic. Other studies reported that both VFC and state laws requiring indemnity insurers to cover childhood immunizations contributed to the decrease in proportion of physicians who referred children to public vaccine clinics for immunizations, where free vaccines were available [7-9]. To investigate the progress on economic barriers, we sought to compare post-VFC data with pre-VFC data. Specifically, given our previous data on reported out-referral in Minnesota and Pennsylvania prior to the VFC program [10-12], we determined the reported out-referral of children by insurance status now, a decade later.

## Methods

We conducted a mailed survey of physicians and obtained doses administered data from the public sector.

### Subjects

For the 2003 survey, we recruited primary care physicians in Minnesota and Pennsylvania who participated in a previous survey on vaccine economics in 1991 or 1993 (State dependent) [10-12]. Minnesota physicians were initially sampled systematically based on a random start point from the Minnesota Medical Association (MMA) master list using four strata: general practitioners (GPs), board-certified family physicians (FPs) in urban and suburban areas, board-certified FPs in rural areas, and board-certified pediatricians (Peds). The initial Pennsylvania physicians came from a random sample of GPs, FPs, and Peds from the combined listings of the American Medical Association and the American Osteopathic Association [11].

We surveyed 340 primary care physicians in 2003 from Minnesota and Pennsylvania, of whom 26 were determined ineligible because they had retired, died, no longer saw children, or moved out of state. Of the remainder, 118 could not be reached, and 22 refused, leaving 174 physicians. We excluded 10 physicians who returned surveys anonymously. Our sample in 2003 was thereby reduced to 164 physicians (89 from Minnesota and 75 from Pennsylvania) who completed the survey for a response rate of 54% (164/304); the refusal rate was 7%. All 164 physicians were surveyed previously in 1991 or 1993. Before the study, power calculations determined that the sample size required for the matched pair analyses to detect a difference of 10% (0.5) based on a mean response of 5 with a standard deviation of 2.0 on a scale of 0 to 10 was at least 126 respondents to have statistical power of 0.80 with a type I error rate of 0.05. Unfortunately, we were unable to test for demographic differences among physicians over time (i.e., 1991 or 1993 vs. 2003 data) because we were unable to access data from 1991 or 1993.

### Questionnaire

We chose PRECEDE-PROCEED, a systematic process to evaluate health problems and design intervention programs [13,14], as the framework for concepts on the questionnaire. This framework allowed us to include various barriers and facilitators to vaccination, including economics and insurance. For many questions, respondents were asked to rate on a scale of 0 (very unlikely) to 10 (very likely) how likely they were to recommend immunization for a child in a particular clinical situation (e.g. uninsured, Medicaid, or insured child). The questionnaire was pilot-tested and subsequently revised [15].

### Data collection

Data were collected by questionnaires mailed in three waves; data from completed questionnaires were entered twice and compared to reduce data entry errors. Non-respondents to the third wave were contacted by trained interviewers using computer-assisted telephone interviewing (CATI) which allowed direct data entry during the interview [16], eliminated unintentionally skipped questions, and provided automatic range and logic checks. Data collection began in January 2003 and continued into August 2003. Participants were offered a $30 honorarium. The Institutional Review Board of the University of Pittsburgh, Olmsted Medical Center, and the Centers for Disease Control and Prevention approved this project.

### Statistics

For a clearer presentation of the data and due to our modest sample, the likelihood of referring children to the public health department for immunizations which was rated on scales of 0–10, was collapsed into the categories: "unlikely" (rating 0–5) and "likely" (rating 6–10). This categorization was chosen because of the bimodal distribution. Changes in the number of referrals since 1993 and the impact programs had on referral to public vaccine clinics were collapsed into "decrease" (rating 0–3) "no change" (rating 4–6) and "increase" (rating 7–10). Adequacies of Medicaid were rated on a scale of 0–10 were collapsed into "inadequate" (rating 0–3), "intermediate adequacy" (rating 4–6), and "adequate" (rating 7–10). Chi-square tests for associations were performed on the contingency tables. Student's t tests were performed to test for group differences of continuous variables. Paired t tests were used to test for pre- versus post-VFC differences in physician ratings. Multiple linear regression analyses to determine which variables predict physician likelihood of referral to public vaccine clinics did not contribute additional information already obtained from the bivariate analyses. Therefore, we did not report these models. All statistical analyses were achieved by using SAS 8.2 statistical software (SAS Inc, Cary, North Carolina). Statistical significance was set at *P *≤ 0.05.

## Results

### Demographics

The demographic characteristics of Minnesota and Pennsylvania physicians were generally similar (Table 1). However, Minnesota respondents saw a somewhat lower percentage of primary care patients than Pennsylvania physicians (96% vs. 98%, *P *= 0.007).

### Impact of insurance status on referral to health department clinics for immunizations

Physicians were asked in 2003 to rate their likelihood on a scale of 0 (very unlikely) to 10 (very likely) of referring a child to the health department for immunization in a series of survey questions in which only the insurance status of the patient changed. The percentage of respondents likely to refer was 60% for an uninsured child whose parents are unable to pay, 50% for a child with insurance that did not cover vaccines, 14% for a child with Medicaid, and 3% for a child with insurance that pays for immunization. There were no state differences in referring Medicaid-insured children, children with insurance that pays for immunization, and children whose insurance did not pay for vaccine; however, Minnesota physicians (68%) reported being more likely than Pennsylvania physicians (51%) to refer uninsured children whose parents were unable to pay (*P *= 0.024).

The majority of physicians (57%) reported a higher likelihood of referring an uninsured child as compared with a child with insurance coverage to a health department vaccine clinic; 65% of these physicians participated in VFC and 16% did not know about their VFC participation. Of those physicians who would not refer either child (38%), the majority (87%) participated in VFC and 5% did not know about their VFC participation. Only 2% of physicians reported that they would refer both insured and uninsured children for vaccination.

### Participation in VFC and impact on referral to health department for immunization

**Figure 1:**
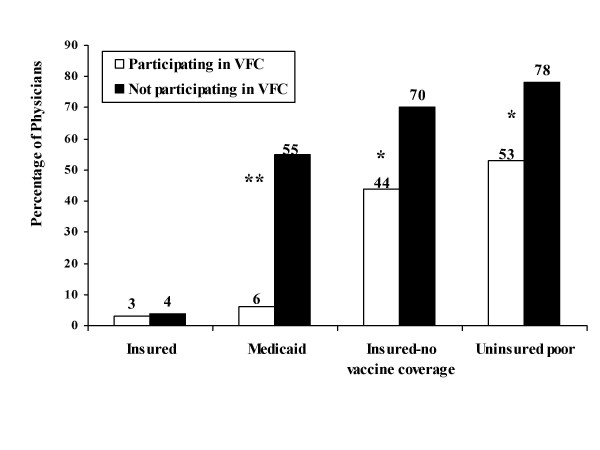
Percentage of physicians in 2003 likely to refer children to public health department vaccine clinics by participation in VFC and by patient insurance status. *Vaccine*, Zimmerman, RK, et al. Legend: Likely is defined as 6 to 10 on a scale of 0 to 10. ** P ≤ 0.001 * P ≤ 0.05

The proportions of Minnesota and Pennsylvania physicians, respectively, participating in VFC was 71% and 76%, nonparticipating was 11% and 19% and did not know was 17% and 5% (*P *= 0.041). More physicians who did not participate in VFC were likely to refer children to the health department for immunizations than those who participated (Figure 1). The difference was especially marked with regard to Medicaid children, for whom 55% of the physicians who did not participate were likely to refer as compared with 6% of those who participated in VFC (*P *< 0.001).

When state comparisons were made, similar results were found for referral of insured and insured without vaccine coverage; however state differences were apparent for Medicaid-insured and uninsured children. Comparing physicians who participated and did not participate in VFC, the likelihood of out-referring a Medicaid-insured child was 5% versus 77% (*P *< 0.001) in Pennsylvania and 6% versus 22% (*P *= 0.123) in Minnesota. Comparing physicians who participated and did not participate in VFC, the likelihood of out-referring an uninsured child was 40% versus 86% (*P *= 0.002) in Pennsylvania and 65% versus 67% in Minnesota (*P *= 0.922).

### Determinants of self-reported change in out-referral

Almost a quarter (24%) of physicians reported decreased referrals of children to public vaccine clinics in a question about changes in the number of referrals since 1993. The impact being greater in Pennsylvania than Minnesota (P < 0.01; Table 2). The VFC program and Children's Health Insurance Program (CHIP) are associated with changes in reported referral since 1993 (Table 2).

Over a third of physicians (34%) reported that the VFC program decreased the number of referrals from their practice to public vaccine clinics, 7% reported increased referrals, and 60% gave intermediate responses. The impact differed by state: 23% of Minnesota physicians reported decreased referrals because of VFC compared with 45% for Pennsylvania physicians (P = 0.003).

Almost a quarter (24%) of physicians reported that CHIP decreased the number of such referrals, 7% reported increased referrals, and 69% gave intermediate responses. This also varied by state: 15% of physicians in Minnesota reported decreased referrals attributed to CHIP compared with 35% of physicians in Pennsylvania (P = 0.003). In Pennsylvania, 27% of physicians reported that the "first dollar" law decreased the number of referrals, 10% reported increased referrals, and 63% gave intermediate responses.

### Medicaid issues

Few physicians (12%) thought that the reimbursement by traditional Medicaid for the vaccine administration fee was adequate; 51% thought it was inadequate, and 37% gave intermediate answers. Perceptions of the adequacy of this administration fee did not vary by state. Even fewer physicians (10%) felt that the monthly capitation rate from Medicaid HMOs was adequate; over half (56%) rated it as very inadequate, and over a third (34%) gave intermediate responses. Adequacy of the monthly capitation rate from Medicaid HMOs did vary by state with more Pennsylvania physicians reporting that it was inadequate than Minnesota physicians (69% vs. 45%, *P *= 0.005). Neither the perceived adequacy of the Medicaid vaccine administration fee nor the adequacy of the monthly capitation rate was related to the likelihood of referral of a Medicaid-insured child.

### Historical comparisons of out-referral before and after VFC

Comparisons of physician likelihood to out-refer using data from the surveys conducted before VFC and after VFC implementation revealed a significant drop in the likelihood to out-refer. Physician likelihood to refer an uninsured child for vaccination, measured on a scale of 0 to 10 points where 10 is very likely, decreased by a mean difference of 1.9 points (*P *< 0.001) from pre- to post-VFC. The likelihood to refer a Medicaid-insured child decreased by a mean of 1.2 points (*P *= 0.001). For an insured child, there was mean decrease of 0.7 (*P *= 0.01). When these data are stratified by state, there was a decrease in the likelihood of MN physicians to refer a Medicaid insured child by a mean difference of 0.7 points (*P *= 0.05). Among PA physicians, we found a decrease in their likelihood of referring an uninsured child for vaccination by 3.8 points (*P *< 0.001) and a decrease in their likelihood of out-referrals for Medicaid insured children by 2.5 points (*P *< 0.001).

## Discussion

To our knowledge, this is the only study of vaccine economics over a decade that had baseline data from interviews conducted before the VFC and could directly match individual responses for a true pre-post comparison.

We found that physicians' reported likelihood of referring children to public vaccine clinics decreased from before VFC to after its implementation and that VFC and CHIP were credited for this decrease. Differential referral based on insurance status continues to occur, especially for clinicians who are not participating in VFC. Clinicians who are participating are much more likely to vaccinate Medicaid and uninsured children in their office. Previous reports attributed decreased out-referrals of children for immunizations to the VFC [7,17], to changes in state insurance coverage laws [7,17], and to a universal vaccine purchase program [18]. Moreover, inner city physicians generally attributed increased immunization rates to the VFC [19].

Generally, Minnesota and Pennsylvania have progressive immunization financing systems. A first dollar immunization coverage law was implemented in 1988 in Minnesota and in 1992 in Pennsylvania that enables insured children to receive vaccines at their private physicians' offices. The VFC program was implemented on October 1, 1994 in Minnesota, on January 1, 1995 in Philadelphia, and April 1, 1996 in Pennsylvania generally. The first dollar laws greatly reduce under-insurance for immunization; however, certain plans are exempt [i.e., ERISA (Employee Retirement Income Security Act) plans]. The children who remain under-insured can be vaccinated in their medical home in Minnesota, using vaccine typically purchased by the state; in Pennsylvania, the under-insured can be referred for vaccination to public health vaccine clinics or federally qualified health centers where VFC vaccine can be used for the under-insured. Despite these differences, we found no difference between the states in out-referral of under-insured children which we speculate is due to the success of first dollar laws, the limited number of ERISA plans, or good vaccine coverage with ERISA plans.

**Figure 2:**
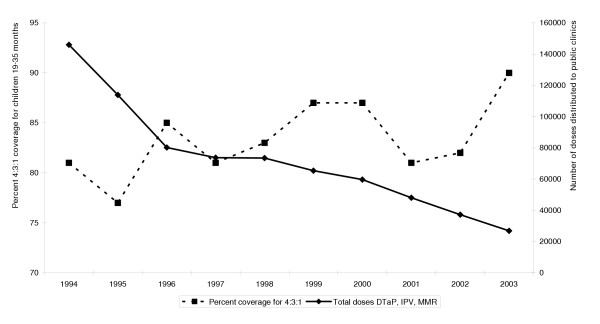
Total doses DTP, polio and MMR vaccines administered at Minnesota health departments and overall 4:3:1 vaccine coverage rates for Minnesota, 1994–2002. *Vaccine*, Zimmerman, RK, et al.

**Figure 3:**
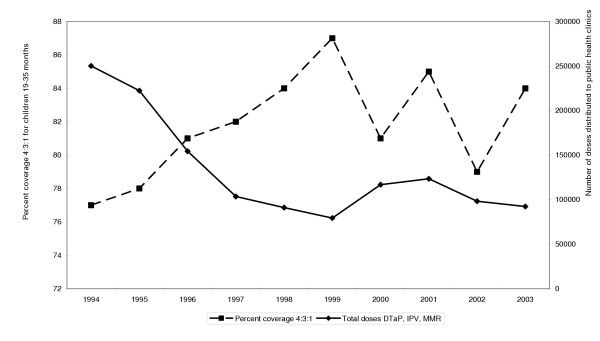
Total doses DTP, polio and MMR vaccines administered at Pennsylvania health departments and overall 4:3:1 vaccine coverage rates for Pennsylvania, 1994–2002. *Vaccine*, Zimmerman, RK, et al.

Given the billion dollar cost of VFC, it is critical to have data to collaborate our survey data. In the current study, we report substantial decreases in doses administered by public health agencies in MN and PA since 1993 [See Figures 2 and 3], [see Additional file 1]. Because overall immunization rates have been increasing, the decline in vaccinations given by public health agencies is consistent with increased vaccinations given by private clinicians. Szilagyi et al [20], found that the number of childhood vaccinations delivered at health department clinics in PA declined 56% between 1993 and 1997 in PA.

A possible explanation for decline in number of vaccine doses administered by health departments [See Additional file 1] may be decreases in public health staffing due to either federal Section 317 or state funding reductions. PA began examining the transfer of some state health clinic functions including immunizations, in 1996. The study's authors reported that immunization services could be as effectively delivered by the private sector as by public health departments in both urban and suburban areas, but not in rural areas [21]. However, this demonstration project was neither large enough, nor early enough to explain observed declines in public sector vaccinations in PA.

### Outcome of referral of disadvantaged children to public vaccine clinics

Although referral of children from primary care physician's offices to public vaccine clinics is preferable to not vaccinating, there are disadvantages. First, the child may visit the public vaccine clinic late and therefore have a window of time when they are not age-appropriately vaccinated and therefore are susceptible to vaccine-preventable diseases. Second, fragmentation of care occurs with an increased burden and expense for the parents of taking the child to one site for vaccines and another site for well-child care and other services. A study after VFC implementation found that 73% of children received all or some immunizations within a medical home [22]. Another study found that the percentage of children up-to-date with DTaP was 2%-9% lower when comparing records from the most recent provider with a fuller set of records [23]. Third, until wider use of immunization registries, medical records are not easily transferred from one site to another and therefore each site may lack important information available at the other.

### Limitations

This study has certain limitations. The response rate was modest. It is impossible to know to what extent, if any, the non-respondents differ with regard to their current demographics, attitudes and practices concerning vaccine economics. By design, this sample represents physicians, who have been in practice over a decade, not all physicians in current practice. Arguably, the results cannot be generalized beyond the states of Minnesota and Pennsylvania; however, the 2002 4:3:1:3:3 immunization series (e.g., of 4 doses of DTP, 3 doses of poliovirus vaccine, 1 dose of measles containing vaccine, 3 doses of Hib, 3 doses of Hepatitis B vaccine) completion rates in these two states were 77% and 75%, respectively, which are fairly close to the national rate of 75% [24]. In addition, these two states represent two very different socio-political environments, one a predominantly rural, large, midwestern region and the other, a more urban, Eastern region. Finally, before-and-after studies on the same individuals identify changes but do not necessarily prove the reason for the changes. Because several policy and programmatic changes occurred during this time period, ability to attribute changes to any one program may be limited. However, the addition of doses administered in the public sector greatly strengthens the conclusions.

## Conclusion

Reported out-referral by primary care physicians to public vaccine clinics decreased from 1991–1993, that is before VFC, to this 2003 study. Fewer doses being administered at health departments coupled with higher rates of immunizations, suggest that more vaccines were being administered in private provider offices.

## Competing interests

The author(s) declare that they have no competing interests.

## Authors' contributions

Richard Kent Zimmerman, MD, MPH: Funding, design, data interpretation, manuscript writing

Melissa Tabbarah, PhD, MPH: Data analyses, manuscript writing

Janine E. Janosky, PhD, Advice on data analyses and interpretation/ senior statistician

Barbara Bardenheier, MPH, MA, Epidemiologic advice, data interpretation, manuscript editing

Judith A. Troy, MS, Data collection, manuscript assistance

Ilene K. Jewell, MS Hyg, Instrument design, data collection

Barbara P. Yawn MD, Msc. Assistance with recruitment, interpretation of data

## Pre-publication history

The pre-publication history for this paper can be accessed here:



## Supplementary Material

Additional File 1 "Methods: Vaccine doses administered in the public sector" and "Results: Doses Administered". Minnesota and Pennsylvania state health departments provided data on doses of diphtheria-tetanus-pertussis (DTP or DTaP), poliovirus, and measles-mumps-rubella vaccines (MMR) administered in their respective states for the years 1994–2003. Immunization rates for these states for the years 1994–2003 were obtained from the National Immunization Survey. These data are graphed by year in Figures 2 and 3. Click here for file

## Figures and Tables

**Table 1:** Demographic characteristics of physician respondents by state

Variable	Minnesota (n = 89)	Pennsylvania (n = 75)
Gender (% male)	75	71
Specialty		
Family Practitioner/General Practitioner	51	47
Pediatrician	49	53
Number of primary care physicians in practice (%)		
0–2	36	26
3–5	27	29
6+	37	45
Number of patients under age 6 years seen in a typical week, (mean ± SE)	60.6 (15.4)	42.8 (3.8)
Percentage of primary care patients, (mean ± SE)*	96 (0.8)	98 (0.5)
Percentage of patients with payment type		
Self pay or uninsured (mean ± SE)	6 (0.7)	5 (0.7)
Medicaid HMO (mean ± SE)	11 (1.9)	12 (2.7)
Commercial HMO/PPO (mean ± SE)	40 (3.4)	40 (2.7)
Fee for service insurance (mean ± SE)	18 (2.6)	20 (2.5)
Traditional Medicaid (mean ± SE)**	7 (1.3)	4 (1.1)

Means are reported with standard error (SE).

*P < 0.01 by t test.

**P < 0.05 by t test.

**Table 2:** Change in reported referrals to public vaccine clinics from 1991/1993 to 2003, by perceived impact of VFC and CHIP, on referrals

Variable	How has the number of child referrals to public vaccine clinics changed since 1993			
	
	Decrease (%)	Same (%)	Increase (%)	Total (%)
Overall	24	66	10	100
State*				
Minnesota	18	76	6	100
Pennsylvania	31	55	15	100
Impact of VFC on referrals*				
Decrease	62	25	13	100
No change	1	93	6	100
Increase	36	36	27	100
Impact of CHIP on referrals*				
Decrease	55	32	13	100
No change	11	84	5	100
Increase	36	27	36	100

* *P *< 0.01 by X^2 ^test.
