# Supplementary material for: Impact of vaccine economic programs on physician referral of children to public vaccine clinics: a pre-post comparison
Source: BMC Public Health. 2006 Jan 12;6:7. doi: 10.1186/1471-2458-6-7 (PMC1388204; doi:10.1186/1471-2458-6-7)
Supplement: Additional File 1 — "Methods: Vaccine doses administered in the public sector" and "Results: Doses Administered". Minnesota and Pennsylvania state health departments provided data on doses of diphtheria-tetanus-pertussis (DTP or DTaP), poliovirus, and measles-mumps-rubella vaccines (MMR) administered in their respective states for the years 1994–2003. Immunization rates for these states for the years 1994–2003 were obtained from the National Immunization Survey. These data are graphed by year in Figures 2 and 3. [file 1471-2458-6-7-S1.doc]

**Additional File 1**

**Methods: Vaccine doses administered in the public sector**

Minnesota and Pennsylvania state health departments provided data on doses of diphtheria-tetanus-pertussis (DTP or DTaP), poliovirus, and measles-mumps-rubella vaccines (MMR) administered in their respective states for the years 1994-2003. For Minnesota, the data included vaccine doses administered by county public health agencies and Indian Health Service clinics (Lisa Harris, 2004, personal written communication). For Pennsylvania, vaccine data were combined for doses administered at all state health centers and county/municipal health departments. (Nancy Mumper, 2004, personal written communication).

Immunization rates for these states for the years 1994-2003 were obtained from the National Immunization Survey (NIS, www.cdc.gov/nip/coverage/default.htm#chartz), a telephone survey from which immunization data are verified by checking with the child’s provider.

**Results: Doses Administered**

The combined total doses of DTP, poliovirus and MMR vaccines administered in health departments in each state is graphed by year (solid lines, Figures 2 and 3). The overall vaccination rate for 4 doses of DTP, 3 doses of poliovirus vaccine and 1 dose of measles containing vaccine (4:3:1) is also graphed by year (dotted lines, Figures 2 and 3). In both states, there is a clear decline in the number of total doses of the 4:3:1 vaccines administered in the public sector from 1994 to 2003, while the percent of children vaccinated increased over that period. Fewer doses being administered at health departments coupled with high rates of immunizations, suggest that more vaccines were being administered in private provider offices.
